# Antimicrobial, antioxidant, toxicity and phytochemical assessment of extracts from A*cmella uliginosa*, a leafy-vegetable consumed in Bénin, West Africa

**DOI:** 10.1186/s12906-016-1014-3

**Published:** 2016-01-27

**Authors:** Latifou Lagnika, Abdou Madjid O. Amoussa, Rafatou A. A. Adjileye, Anatole Laleye, Ambaliou Sanni

**Affiliations:** 1 Unité de Biochimie et Biologie Moléculaire, Equipe de Biochimie et Substances Naturelles Bioactives, Faculté des Sciences et Techniques, Université d’Abomey-Calavi, Cotonou, 04 BP 0320 Bénin; 2 Unité de Biologie Humaine, Laboratoire de Cytogénétique et de Biologie Moléculaire, Faculté des Sciences de Santé, Université d’Abomey-Calavi, Cotonou, Bénin

**Keywords:** *Acmella uliginosa*, Antifungal, Antibacterial, Antioxidant, Toxicity, Phytochemical

## Abstract

**Background:**

*Acmella uliginosa* (Asteraceae) is a flowering plant whose leaves are consumed as a vegetable in Benin. They are also traditionally used as an antibiotic in the treatment of infectious diseases. To evaluate the therapeutic potential and toxicity effect of this leafy-vegetable, the antibacterial, antifungal, antioxidant activities and, toxicity and phytochemical constituents were investigated.

**Methods:**

Dichloromethane, methanol and aqueous extracts of *Acmella uliginosa* were evaluated for their antimicrobial activity against six bacterial and six fungi strains. Antibacterial and antifungal activities were investigated by microdilution method and agar diffusion method respectively. Antioxidant activity was assessed using the 2,2-diphenyl-1-picryl-hydrazyl assay and phytochemical screening was carried out using standard procedures. Finally, oral acute toxicity at a dose of 2000 mg/kg was done according to the Organization for Economic Co-operation and Development guideline n° 423.

**Results:**

The antibacterial activity was broad spectrum, inhibiting both Gram-positive and Gram-negative bacteria. The minimum inhibitory concentration ranged from 0.625 to 5 mg/ml. The antifungal evaluation show that all the extracts inhibited mycelial growth and sporulation of fungi with percentages of inhibition ranging from 9.39 to 75.67 % and 22.04 to 99.77 %, respectively. In DPPH radical scavenging assay, the effect on reducing free radicals increased in a dose dependent manner. The percentage of inhibition of DPPH ranged from 0.94 to 73.07 %. Phytochemical screening revealed the presence of coumarin, flavonoid, naphtoquinone, anthracene derivative, saponin, lignan, triterpene and tannin. The dichloromethane and methanol extracts showed the best biological activities; they were also shown as the best extraction solvents of phytochemicals. In the acute toxicity evaluation, all animals were physically active and no deaths of rats were observed during the test. However, the aqueous extract promoted biochemical, hematological and histopathological alterations of treated rats at 2000 mg/kg body weight.

**Conclusion:**

*A. uliginosa* extracts contains antimicrobial, antioxidant agents and was not lethal for rats when ingested. However, according to the results obtained for biochemical, hematological, and histopathological analysis, caution is required regarding its consumption.

## Background

The nutrition and health of the world population are the main upcoming challenges particularly in developing countries. In Sub Saharan Africa, there are more than 45,000 plant species of which about 1000 can be consumed as leafy vegetables which happen to be the mainstay of traditional African diets [1]. Leafy vegetables are plant species whose leaves are used as a vegetable in the sauce preparation. They play a very important role in our diet and are the most readily available sources of carbohydrates, fats, proteins, vitamins, minerals, essential amino acids [2, 3]. Many leafy vegetables are mainly consumed for their nutritional values since immemorial times without much consideration for their medicinal importance. Apart from their nutritional intake, they have the ability to synthesize several secondary metabolites of relatively complex structures possessing antioxidants [4]. These metabolites produce specific effects on the physiology of human being and other organisms. Recent reports indicate that there is an inverse relationship between the dietary intake of antioxidant rich foods and the incidence of human diseases [5]. The interest on these leafy vegetable has increased as a result of epidemiological studies linking eating habits and prevalence of certain diseases. Previous research indicated antimicrobial [6, 7], antidiabetic [8], anti-histaminic [9], anti-carcinogenic and hypolipidemic [10, 11] properties of leafy vegetables. Leafy vegetables are popular in Benin but there is no scientific data available on their medicinal properties and toxicity.

*Acmella uliginosa* (Sw.) Cass. (Asteracea) is a species of flowering plant which is indigenous and widely distributed in the tropics and sub-tropics especially in the West Indies, Venezuela, Brazil, Africa, Indonesia and Malaysia [12]. It is found in the North-west of Benin specifically in Atacora region [13]. *Acmella uliginosa* is commonly used by the Malay community in Malaysia to relieve pain often associated with mouth ulcers, toothache, sore throat, and stomach ache [14]. The methanolic flowers extract was reported to display potent antinociceptive property [15]. In Benin it is a traditional leafy vegetable which has been domesticated in rural areas and whose sauce is a good dewormer and antibiotic [13, 14]. Aside these reports, and to the best of our knowledge, no other pharmacological effects of this plant related to its traditional use as antibiotic have been reported. Therefore, the objectives of the present study were (1) to evaluate the antimicrobial and antioxidant properties of the extracts of *Acmella uliginosa* leaves; (2) to determine the toxicity of aqueous extract of *A. uliginosa* using an acute oral toxicity test in animal models.

## Methods

### Plant collection

The leaves of *A. uliginosa* were collected in September 2012 from the airport garden of Cotonou, department of Littoral, Southern Benin. Identification of the specie was carried out by botanists from the University of Abomey-Calavi and a voucher specimen (AA6624/HNB) was deposited at the National Herbarium of Bénin. After identification, the collected leaves were ground using an electric grinder (MARLEX Electroline Excella).

### Extracts preparation

The leaves of *A. uliginosa* (620 g) were successively extracted by maceration with 1000 ml of dichloromethane (DCM) and 750 ml of methanol (MeOH) for 72 h stirring while a second extraction (decoction) with 1000 ml of sterile distilled water (H_2_O) was carried out with five hundred grams (500 g) of plant material. Each extraction was repeated three times. The filtrates of each extraction were desiccated under vacuum and the obtained extracts were stored at 4 ° C until biological assay.

### Phytochemical

Phytochemical screening of the plant was carried out according to the standards methods for the detection of plant secondary metabolites [16, 17]. Alkaloids, flavonoids, steroids, coumarins, saponins, naphthoquinones, triterpenes, lignans, pigments, anthracene derivatives were investigated using TLC method [16] while tannins were characterized using iron-III-chloride reagent [17].

### Antibacterial activity

#### Bacterial strains

Dichloromethane, methanol and aqueous extracts of *A. uliginosa* were individually tested against a panel of bacteria including four Gram-positive: *Staphylococcus aureus* (ATCC 6538), *S. epidermidis* (CIP8039), *Enterococcus faecalis (*ATCC 29212), *Staphylococcus aureus* Methicillin Resistant (SARM) and two Gram-negative: *Escherichia coli* (CIP 53126) and *Pseudomonas aeruginosa* (CIP82118) obtained from Laboratoire de Biophotonique et Pharmacologie, University of Strasbourg, France.

#### Growth inhibition effect of extracts at 10 mg/ml

Sensivity of different bacterial strains to various extracts was determined using 96-well microplate. The aim of this method was to eliminate the extracts, which at 10 mg/ml do not inhibit the growth of bacteria [18]. The extracts were reconstituted to a concentration of 20 mg/ml in acetone/Muller Hinton broth culture. A volume of 100 μl of each extract (20 mg/ml) was introduced in triplicate microplate already seeded with 100 μl of the Muller Hinton broth culture inoculums (10^6^ CFU/ml) of the tested bacteria. The microplate was incubated at 37 °C. After 18 h of incubation, 40 μl of 0.2 mg/ml solution of *p*-iodonitrotetrazolium (Sigma Aldrich) were added to each well and microplate was incubated at 37 °C. Finally, after 30 min, the color change (extract color to red) of mix in each well was examined to select actives extracts. Active extract do not change color.

#### Minimum inhibitory concentration and total activity

The Minimum inhibitory concentrations (MIC) of selected extracts were determined by the method of broth microdilution using *p*-iodonitrotetrazolium (INT) as an indicator of bacterial viability [19]. Briefly, 100 μl of Mueller Hinton broth (DIFCO) were added to each well of a 96-well microplate and 100 μl of extracts (20 mg/ml) were added to the first well (A) of the plate. A two-fold dilution was carried to make 8 concentrations. Then, 100 μl of bacterial broth at 10^6^ CFU/ml were finally added into all the wells. After 18 h incubation at 37 °C, 40 μl of *p*-iodonitrotétrazolium (0.2 %) were added to each well and the incubated was continued at 37 °C. After 1 h incubation, the MIC values were recorded. Gentamicin was used as positive control. Each assay was run in triplicate. The total activity of each extract was calculated by dividing the MICs with the amount of extract obtained from 1 g of plant material [20]. This value indicates the volume in which the active extract obtained from 1 g of dry plant material can be diluted to always have inhibitory activity against organisms [21].

### Antifungal assay

#### Test organisms

Fungi strains used in this study included *Aspergillus flavus* CMBB75, *A. parasiticus* CMBB20, *A. ochraceus* CMBB91, *A. nidulans* CMBB90, *A. clavatus* NCPT24 and *A. fumigatus* CMBB89. They were obtained from the laboratory of biochemistry and molecular biology at the University of Abomey. These microorganisms are the most common fungal pathogens of vegetables, animals and humans. They play an important role in opportunistic infections in immunocompromised patients [22].

#### Antifungal test

The in vitro antifungal activity of extracts was evaluated on mycelia development and sporulation as described previously by Dohou et al. [23] with minor modifications. 10 ml of the mixture of potato dextrose agar-extract at 1 mg/ml were poured into sterile petri dishes. Fungi suspension were prepared in tween (5 %) and 100 spores were dropping in the center of petri dishes. After 5 days incubation at 25 °C, the diameter of mycelia was measured and the number of spores was counted microscopically using Malassez cell. Each test was performed in triplicate. Three petri dishes containing potato dextrose agar without extract were used as negative control and Fluconazol (100 μg/ml) was used as positive control. The percentage of inhibition (PI) of extracts was determined according to the formula below:

$$ \mathrm{PI}\ \left(\%\right) = \kern0.5em \frac{{\mathrm{A}}_{\mathrm{v}\ \mathrm{control}}\hbox{-}\ {\mathrm{A}}_{\mathrm{v}\ \mathrm{tested}\ \mathrm{extract}}}{{\mathrm{A}}_{\mathrm{v}\ \mathrm{control}}}\kern0.5em \mathrm{x}\ 100 $$

In which A_v control_ = average diameter of the mycelia or estimated number of spores of control (*n* = 3)_,_ A_v tested extract_ average diameter of the mycelia or estimated number of spores of tested extracts (*n* = 3).

### DPPH radical-scavenging activity

The ability of the extracts to scavenge the DPPH (2,2-diphenyl-1-picrylhydrazyl) radical was evaluated. The antioxidant activity was determined according to the method previously described by Velazquez et al. [24]. The stock solution of the extracts was prepared at 1 mg/ml. Then, a two-fold serial dilution was carried to make 8 concentrations (1 - 0.007 mg/ml). Briefly, 1.5 ml of a freshly prepared methanolic solution of DPPH (2 %) was mixed with 0.75 ml of extract solution. After 15 min incubation in the dark, at room temperature, the absorbance of the mixture was read at 517 nm using a spectrophotometer (Jenway, Genova). The blank consisting of a mixture of 1.5 ml of methanol and 0.75 ml of extract solution. Quercetin was used as positive control. All assays were performed in triplicate. The percentage of inhibition of DPPH radical was calculated according to the following formula:

$$ \%\ \mathrm{inhibition} = \kern0.5em \frac{{\mathrm{A}}_{\mathrm{blank}}\hbox{--}\ {\mathrm{A}}_{\mathrm{sample}}}{{\mathrm{A}}_{\mathrm{blank}}}\kern0.5em \mathrm{x}\ 100 $$

A_blank_ = absorbance of blank_,_ A_sample =_ absorbance of tested extract

### Oral acute toxicity

#### Experimental animals

Six female wistar rats with body weight ranged from 180-200 g were obtained from the Laboratoire de Biologie Humaine, Faculty of Medicine, University of Abomey-Calavi. The used animals were nulliparous and non-pregnant. The rats were fed with standard laboratory diets, given water *ad libitum* and maintained under laboratory conditions (22 ± 3 °C), a relative humidity between 30-70 % and a constant light-dark schedule (12 h light/dark cycle).

#### Oral acute toxicity testing

The toxicity of the aqueous extract of *A. uliginosa* was evaluated according to the Organization for Economic Co-operation and Development guidelines n° 423 [25]. The protocol related to acute toxicity test was approved by the scientific committee of research protocols (VPMAS/PFCR-2/UAC), University of Abomey-Calavi, Bénin. A total of six females rats were divided into two groups with three animals each and kept in different cages for easy observation during experiment. Distilled water (10 ml/kg body weight) was given to control group (group I). The group II was given a single dose of the aqueous extract solution (2000 mg/kg body weight). Following administration of extract, rats were closely monitored for 30 min and 2, 4, 8 and 24 h. Mortality, food and water consumption and general acute toxicity or clinical symptoms were recorded. Body weight was also recorded on days 1, 7 and 14.

#### Haematological and biochemical parameters

At the end of the experiment, all the rats were anaesthetized using thiopental at 0.5 ml/Kg body weight. Animals were then sacrificed and the blood for biochemical and hematological analysis were collected through cardiac puncture into ethylenediaminetetraacetic acid (EDTA) tubes. Each blood sample was analyzed for haematological parameters such as hematocrit (Ht), red blood cells (Rbc), hemoglobin concentration (Hc), mean corpuscular hemoglobin concentration (MCHC), mean corpuscular volume (MCV), mean corpuscular hemoglobin levels (MCH), white blood cells (Wbc), basophils (B), lymphocytes (L), monocytes (M)using an automatic hematological analyzer (Sysmex, XP-300, Japan). Biochemical parameters such as Glucose (GLU), Creatinine (CREA), cholesterol (CHOL), alanine aminotransferase (ALT), aspartate transaminase (AST) were determined using an autoanalyzer (ErbaChem 7, Germany). The liver and kidneys of rats (group I and II) were collected, weighed immediately and transferred to a saline solution. These organs were fixed in 10 % buffered formalin for histological examination. The samples were then treated with increasing concentrations of ethanol and infiltrated with paraffin. Then, the thin cuts were made and stained with hematoxylin and eosin stains.

### Statistical analysis

The *t*-student test was used for statistical analysis of data on body weight, hematological and biochemical parameters. The difference was considered statistically significant when the *p* value was 0.05 or less (*p* < 0.05). All data were expressed as mean ± SD. The graphical representation was performed using the Graph Pad Prism 5.0 software (Microsoft, USA).

## Results

### Phytochemical results

The phytochemical experiments on fresh leaves of *A. uliginosa* revealed the presence of coumarin, flavonoid, naphtoquinone, anthracene derivative, saponin, lignan, triterpene and tannin (Table 1). Dichloromethane extract showed the presence of flavonoid, naphtoquinone, anthracene derivative, lignin and triterpene followed by methanolic extract (Coumarin, flavonoid, naphtoquinone, lignan and triterpene). Aqueous extract revealed coumarin, saponin and tannin. However, the extracts did not show the presence of alkaloid and pigment.

**Table 1:** Phytochemicals screening of extracts from *Acmella uliginosa*

Phytochemicals	Reagents	DCM	MeOH	H_2_O
Coumarin	10 % KOH-EtOH	-	+	+
Flavonoid	NP/PEG	+	+	-
Naphtoquinone	10 % KOH-MeOH	+	+	-
Alkaloid	Dragendorff	-	-	-
Anthracene derivative	5 % KOH-EtOH	+	-	-
Saponin	Vanillin-sulphuric acid	-	-	+
Lignan	Vanillin-phosphoric acid	+	+	-
Triterpene	Anisaldehyde-sulphuric acid	+	+	-
Tannin	Vanillin-sulphuric acid	-	-	+
Pigment	Anisaldehyde-sulphuric acid	-	-	-

DCM dichloromethane extract, MeOH Methanol extract, H_2_O aqueous extract; (+) present, (-) absence

### Antibacterial activity

#### Growth inhibition effect of extracts at 10 mg/ml

Table 2 shows the result of the antibacterial potency of extracts of *A. uliginosa* against selected bacteria. The results showed that the tested microorganisms were more sensitive to the dichloromethane and methanol extracts than the aqueous extract. The dichloromethane extract was the most active by inhibiting the growth of five bacteria strains out of six (5/6). All extracts inhibited the growth of *E. faecalis*. The methanolic extract remains moderately active by inhibiting the growth of *E. faecalis* (Gram-positive) and *P. aeruginosa* (Gram-negative). Unlike other tested bacteria, *E. coli* was resistant to all extracts.

**Table 2:** Antibacterial activity of leaves extracts from *Acmella uliginosa* at 10 mg/ml

	Growth inhibition effect of extracts at 10 mg/ml					
	Gram (+)				Gram (-)	
Extracts	*S.aureus*	*S.a.m.r*	*S. ep epidermidis*	*E. faecalis*	*E. coli*	*P. a*
Dichloromethane	+	+	+	+	-	+
Methanol	-	-	-	+	-	+
Aqueous	-	-	-	+	-	-

(+): actives; (-) : not actives; *S.aureus : Staphylococcus aureus; S.a.m.r : Staphylococcus aureus* meticillin resistant; *S. epidermidis* : *Staphylococcus epidermidis*; *E. faecalis*: *Enterococcus faecalis*; *P. aeruginosa: Pseudomonas aeruginosa*

These results indicated extracts which at 10 mg/ml inhibited (+) or do not inhibit (-) the growth of the microorganisms. Only active extracts at 10 mg/ml will be used for the determination of minimal inhibitory concentration (MIC)

### Minimum inhibitory concentrations (MIC) and total activity

The Minimum inhibitory concentrations (MIC) and total activity of extracts are recorded in Table 3. Several authors have defined concentration ranges to classify the antimicrobial activity of plant extracts and fractions. The activity is high if the MIC is ≤0.1 mg/ml, moderate if 0.1 < MIC ≤ 0.625 mg/ml and weak if MIC > 0.625 mg/ml [21, 26]. Based on these criteria, leaf extracts of *A. uliginosa* showed moderate to low antimicrobial activity with MICs values ranging from 0.625 to 5 mg/ml (Table 3). The dichloromethane extract had moderate activity against all Gram-positive bacteria with a MIC value of 0.625 mg/ml. A MIC of 1.25 mg/ml was obtained with the same extract against *P. aeruginosa* (Gram-negative). The methanolic extract had weak antibacterial activity against *E. faecalis* and *P. aeruginosa* with a MIC of 1.25 mg/ml. The aqueous extract had also shown weak antibacterial activity against *E. faecalis* (MIC = 5 mg/ml). The MIC of gentamicin range from 0.025 to 0.125 mg/ml. The activity of the extracts was still low compared to gentamicin.

**Table 3:** Minimum inhibitory concentration and total activity of selected leaves extracts from *Acmella uliginosa* against tested bacteria

	Minimum inhibitory concentration (mg/ml)				
	Gram (+)				Gram (-)
Extracts	*S. aureus*	*S.a.m.r*	*S. epidermidis*	*E. faecalis*	*P. aeruginosa*
Dichloromethanee	0.625	0.625	0.625	0.625	1.25
Methanol	-	-	-	1.25	1.25
Aqueous	-	-	-	5	-
Gentamicin	0.062	0.062	0.062	0.025	0.125
Total activity (ml/g)					
Dichloromethanee	4.61	4.61	2.30	4.61	2.30
Methanol	-	-	-	23.71	23.71
Aqueous	-	-	-	7.3	-

*S.aureus: Staphylococcus aureus; S.a.m.r: Staphylococcus aureus* meticillin resistant; *S. epidermidis*: *Staphylococcus epidermidis*; *E. faecalis*: *Enterococcus faecalis*; *P. aeruginosa: Pseudomonas aeruginosa*; (-): not tested

The total activity was determined to quantify the antibacterial activity by dividing the mass of extract from 1 g of the plant material by the MIC value. The extracts with higher total activity values are considered the best. The methanol extract had the highest antibacterial activity with a total activity of 23.71 ml against *E. faecalis* and *P. aeruginosa*.

### Antifungal assay

**Fig. 1:**
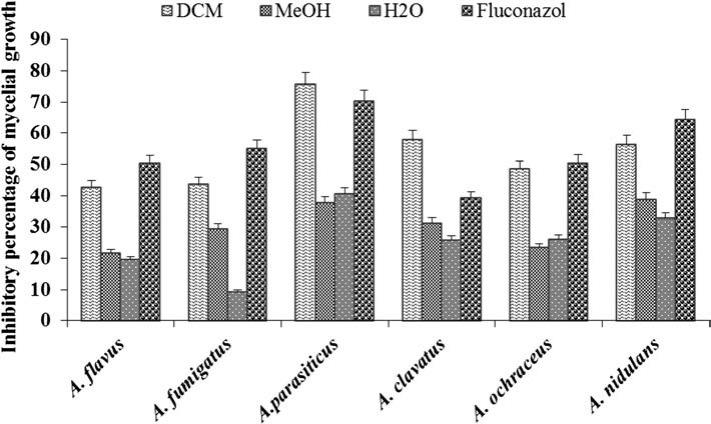
Inhibitory effect of *A. uliginosa* extracts on mycelia growth of A*spergillus* strains. DCM: dichloromethane; MeOH: methanol; H_2_O: aqueous

**Fig. 2:**
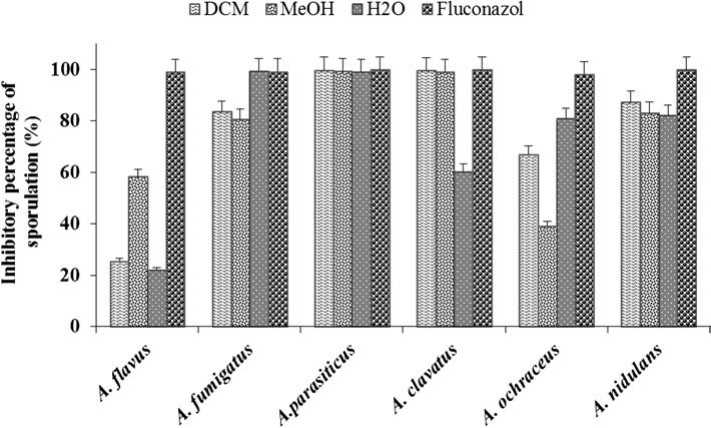
Inhibitory effect of *A. uliginosa* extracts against Aspergillus sporulation. DCM: dichloromethane; MeOH: methanol; H_2_O: aqueous

*Acmella uliginosa* leaves extract showed interesting antifungal activity by inhibiting the sporulation and the mycelia growth of tested fungi (Figs. 1 and 2).

The percentage of inhibition (PI) of extracts on mycelial growth ranging from 9.39 % to 75.67 % (Fig. 1). Only the dichloromethane extract exhibited the strongest activity against *A. parasiticus* with a PI value of 75.67 %. This extract also showed moderate activity against *A. clavatus* (PI = 58.05 %) and *A. nidulans* (PI = 56.38 %). The dichloromethane extract was more active against *A. parasiticus* (75.77 %) and *A. clavatus* (58.05 %) than Fluconazol against *A. parasiticus* (70.27 %) and *A. clavatus* (39.28 %)*.* The other extracts are less active (9.39 % ≤ PI ≤ 40.54 %).

The effect of the extracts at the same concentration is more marked on the inhibition of sporulation than the mycelial growth. Contrary to mycelial growth, the percentage of inhibition of extracts on sporulation of fungi ranged from 22.04 to 99.77 % (Fig. 2). The higher activity was showed by dichloromethane extract against *A. parasiticus* with a PI value of 99.77 % whereas the lowest inhibition was obtained with aqueous extract against *A. flavus* (PI = 22.04 %).

### Antioxidant activity

**Fig. 3:**
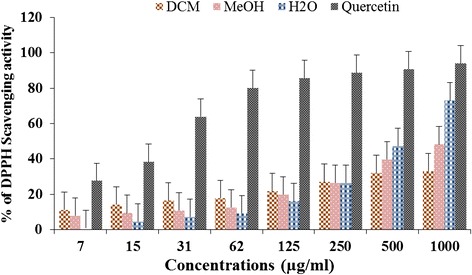
DPPH free radical scavenging activity of *A. uliginosa* extracts. DCM: dichloromethane; MeOH: methanol; H_2_O: aqueous

Each extract was measured for their ability to scavenge DPPH free radicals and results showed on Fig. 3. The results were expressed as percentage of inhibition of DPPH radical in comparison to Quercetin. All extracts showed antioxidant dose dependent activity in a concentration range of 7-1000 μg/ml (Fig. 3). From 7 to 125 μg/ml, the scavenging effects of the extracts decreases in the following order DCM > MeOH > H_2_O. At these concentrations, the percentage of inhibition (PI) varied from 0.94 to 25.96 % compared to the control (27.59 to 88.86 %). At 250 mg/ml the PI of extracts is about 26 % for the three extracts while that of quercetin is 88.86 %. The scavenging effects of extracts at 500 and 1000 μg/ml, increased as follows: DCM < MeOH < H_2_O, and were ranging from 32 to 73.07 %. At the same concentration, the effect of the extracts remains weak compared to quercetin.

### Oral acute toxicity

The oral acute toxicity evaluation showed that animals have tolerated the aqueous extract of *A. uliginosa* leaves at 2000 mg/kg body weight. The tested animals did not display any significant changes in behavioral pattern such as convulsion, diarrhea, salivation, breathing, and impairment in food intake, water consumption, postural abnormalities, hair loss, sleep, restlessness or in physical appearance such as eye color, mucous membrane when compared to the control at the end of 14 days of general observation. No mortality and no visible symptoms of acute toxicity were observed. These results indicated that the LD_50_ value of aqueous extract of *A. uliginosa* is greater than 2000 mg/kg body weight.

**Fig. 4:**
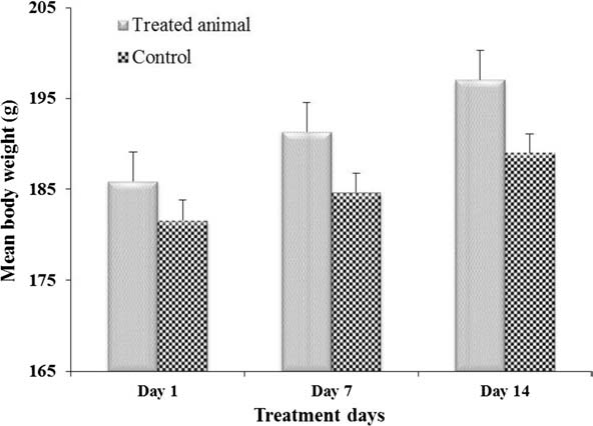
Effect of aqueous extract on Mean body weight of rats for 14 days

**Fig. 5:**
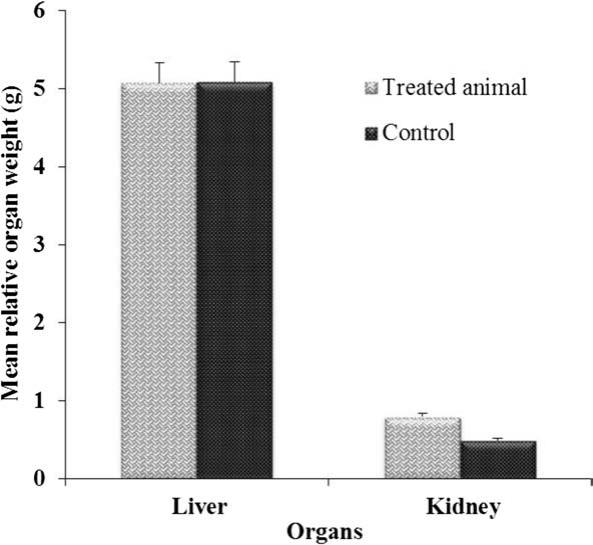
Effect of aqueous extract of *A. uliginosa* (2000 mg/kg) on Mean relative weight of organs in rats for 14 days

**Fig. 6:**
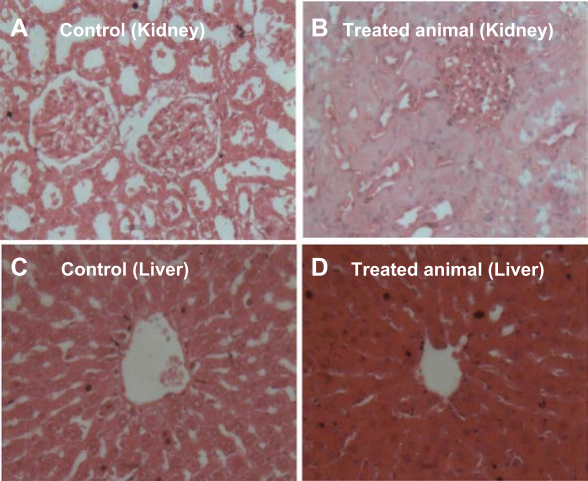
Histological observations of kidney and liver tissues. **a** Kidney of control animal; **b** Kidney of treated animal; **c** Liver of control animal; **d** Liver of treated animal

The body weight of treated rats gradually increased but was not significantly different compared to control (Fig. 4). Similarly, there was no significant difference between the relative liver weight of the test animals and the controls after 14 days of administration of the aqueous extract (Fig. 5). However, a significant increase (*P* < 0.05) was observed in relative kidney weight of treated rats compared to control (Fig. 6).

The hematological parameters were examined in experimental animal after 14 days of administration of the aqueous leaf extracts of *A. uliginosa.* The results are shown in Table 4. Hematological parameters analyzed included the complete blood count of experimental and control group animals. Analysis of blood parameters in animal studies is relevant to evaluate the risk of alterations of the hematopoietic system in toxicity studies, for necessary application to humans [27]. After 14 days of administration of the aqueous extract of *A. uliginosa*, there was a significant increase (*p* < 0.05) of white blood cell count, mean corpuscular volume, mean corpuscular hemoglobin levels and neutrophil count in treated group compared to the control. In the same way, there was a significant decrease (*p* < 0.05) of hematocrit, red blood cell count, lymphocyte and monocyte number in treated groups.

**Table 4:** Effect of aqueous extract of *Acmella uliginosa* on haematological parameters

Haematological Parameters	Experimental	Control
Hc (g/dl)^a^	13.27 ± 0.93	14.13 ± 0.74
Ht (%)^b^	35.33 ± 2.52	43 ± 4
MCV (fl.)^b^	88.33 ± 1.53	70.33 ± 2.52
MCH (Pg)^b^	33 ± 1	23 ± 2
MCHC (%)^a^	37 ± 00	33.33 ± 3.06
Rbc (T/L)^b^	3.99 ± 0.32	6.42 ± 0.60
Wbc (G/L)^b^	6.43 ± 1.50	2.47 ± 0.47
Nc (%)^b^	40.67 ± 8.14	17.33 ± 1.53
L (%)^b^	59 ± 7.94	74.33 ± 0.58
M (%)^b^	0.33 ± 0.58	6.33 ± 2.08

Ht hematocrit, Rbc red blood cells, Hc hemoglobin concentration, MCHC mean corpuscular hemoglobin concentration, MCV mean corpuscular volume, MCH mean corpuscular hemoglobin levels, Wbc white blood cells, Nc Neutrophil count, L lymphocytes, M monocytes. Values are mean ± SEM (*n* = 3 per group), differences were considered significant when *p*-values were less than 0.05 (*p* < 0.05)

^**a**^: values non-significantly different; ^**b**^: values significantly different

Table 5 presents the effects of administration of aqueous extract of *A. uliginosa* on biochemical parameters in experimental rats after 14 days. A significant increase (*p* < 0.05) in creatinine value and a significant decrease (*p* < 0.05) in AST, ALT were observed in treated animals. The values of total cholesterol and glucose did not show any significant change.

**Table 5:** Effects of aqueous extract of *Acmella uliginosa* on biochemical parameters

Biochemical parameters	Experimental	Control
GLU^a^	1.87 ± 0.62	1.34 ± 0.08
CREA^b^	48 ± 7.94	6.3 ± 0.53
CHOL^a^	0.68 ± 0.10	0.68 ± 0.09
AST^b^	152.67 ± 23.29	255.67 ± 10.26
ALT^b^	76 ± 11.79	111.33 ± 44.29

GLU Glucose, CREA Creatinine, CHOL cholesterol, ALT alanine aminotransferase, AST aspartate transaminase. Values are mean ± SEM (*n* = 3 per group), differences were considered significant when *p*-values were less than 0.05 (*p* < 0.05)

^**a**^: values non-significantly different; ^**b**^: values significantly different

Histological examination of visceral organs of treated animals showed a condensation of nucleus, a homogenization and acidophilia of the cell cytoplasm as well as a reduction of capillary spaces in the liver. In the kidneys, histological examination revealed depletion of cortical glomeruli with hyalinization of the cortex and disappearance of urinary room, when compared to the control (Fig. 6).

## Discussion

The therapeutic effects of plant materials generally result from the combination of secondary metabolites. These secondary metabolites are not only essential in the cell structure, but often are involved in the protection of plants against biotic and abiotic stresses. Natural products, as pure compounds or standardized extracts, provide unlimited opportunities for the drug discovery because of the unmatched availability of chemical diversity inside the plants. Phytochemicals are bioactive compound present in leafy-vegetables which could be responsible for their bioactivity linked to the reduced risk of major chronic diseases. It has indeed been estimated that a healthy diet could prevent approximately 30 % of all cancers [28]. The phytochemical screening showed that leaves of *Acmella uliginosa* contain coumarin, flavonoid, naphtoquinone, anthracene derivative, saponin, lignan, triterpene, and tannin. Bioactive compounds are normally accumulated as secondary metabolites in all plant cells but their concentration varies according to the plant parts, seasons, climates, extracting solvent in plant and particular growth phases [29]. Leaves are one of the highest sources of accumulation and are highly beneficial [30].

In this study, dichloromethane extract showed interesting antibacterial (0.625-1.25 mg/ml) and/or antifungal (PI up to 99.77 %) activity by inhibiting one or more microorganisms. These results confirm a statement that the intermediate polarity compounds usually have the highest antimicrobial activity found with many different plant species [31]. The interesting antimicrobial activity of dichloromethane extract against the tested microorganisms could be due to the presence of flavonoids, naphtoquinone and triterpene as reported previously [32–34]. Flavonoids are hydroxylated phenolic substances known to be synthesized by plants in response to microbial infection and they have been found to be antimicrobial substances against wide array of microorganisms in vitro [35]. Flavonoids are known antimicrobial agents throught various mechanisms like inhibition of nucleic acid synthesis, inhibition of cytoplasmic membrane function and energy metabolism [36]. Naphthoquinone and triterpenes have been also reported to possess antibacterial activity [37, 38]. In our study, coumarin and tannin were detected in methanol and aqueous extracts. Coumarins represent a large group of compounds that have been reported to possess a wide range of biological activities including antimicrobial [39] and antioxidant [40]. Tannins are known antimicrobial agents that could inhibit the growth of microorganisms by precipitating the microbial protein and thus depriving them of nutritional proteins needed for their growth and development [41]. This could explain the antimicrobial activity of methanol and aqueous extracts.

DPPH is a stable free radical which accepts an electron or hydrogen radical to become a stable diamagnetic molecule. In the presence of hydrogen doner, DPPH is reduced. It has been showed that the scavenging effects on the DPPH radical increased with the increasing concentration of the samples to a certain extent. In the present study, methanol and aqueous extracts showed appreciable free radical scavenging activity than dichloromethane extract at 500 and 1000 μg/ml. This may be due to the different polarities of antioxidant compounds present in the extracts [42]. The difference in the DPPH radical scavenging activity implies that the extracting solvent would affect the presence of secondary metabolites of extract and then the radical scavenging potency. The antioxidant capacity of methanol and aqueous extracts could be due to the presence of phenolic compounds such as coumarins and tannins. Several researches reported the antioxidant activity of these chemical compounds [43–45].

The oral acute toxicity of aqueous extract at 2000 mg/kg body weight resulted in no mortality and no signs of acute toxicity throughout the 14 days. This suggests that the LD50 is greater than 2000 mg/kg body weight. The increase in body weight observed between day 1 and 14 in the treated groups could be attributed to the increase in food consumption. This could be explained by an excitation of appetite of animal by aqueous extract. In this case, aqueous extract could have hypoglycemia effect. A significant increase (*P* < 0.05) observed in the relative kidney weight of treated animals compare to control could also correlate with the growth of animals or to a dysfunction of kidney cells. Previous studies have shown that a reduction in body weight gain and organ weights is an internal simple and sensitive index of toxicity after-exposure to toxic substances [46, 47]. Contrary to this, the results of our studies showed an increase in body weight and organs of treated rats. Then, it could be concluded that a change in body weight and organs is an index of toxicity.

Hematological assessment is useful to determine the extent of toxic effects of plant extracts on the blood constituents of an animal [48]. In this study, we found a significant difference in hematological parameters such as Rbc, Ht, MCH, MCV, WBC, Nc, L and M. Increase in the production of WBC and it's differentials is generally considered to be a marker of stress and a defense mechanism triggered by immune system against various inflammatory conditions (Polymyalgia rheumatica, bacterial infections, hemorrhage, leukemia etc.) [49]. The significant changes in the level of WBC suggest the toxic effect of the leaves of *A. uliginosa.* Creatinin is known as an effective indicator of kidney function and any significant increase in creatinin levels induces functional nephron damage [50]. The significant rise of creatinine concentration in plasma indicates the implicit effect of the plant on renal filtration mechanism. It was noted a significant reduction of AST and ALT in animals treated compared to the control. This could mean that the aqueous extract of *A. uliginosa* had a harmful effect on the liver. Histological analysis showed lesions in the liver and kidneys. The cellular architecture of these organs confirms the significant changes in hematological and biochemical parameters of treated animals. Coumarins found in *A. uliginosa* extracts are known for their hepatotoxicity and have been reported to be toxic to rats and mice [51]. Thus, this class of compounds may be involved in the changes caused by the aqueous extract of *A. uliginosa.* The lesions observed in the organs could be due to the presence of pesticide residues and/or heavy metal in the leaves of *A. uliginosa*, since the gardener use pesticides and the harvest was made in the garden near Cotonou International Airport. Previous study showed that presence of pesticides in leaf-vegetable could justify the increased kidney weight and increased incidence of chronic nephrosis [52].

## Conclusion

Green leafy vegetables contain various pharmacologically active compounds. The presence of active chemical groups such as flavonoids, coumarin, triterpene, naphtoquinone and tannin in the leaves of *Acmella uliginosa* could justify results obtained. The results of this study confirmed the traditional use of *Acmella uliginosa* as an antibiotic. Leaves extracts of *A. uliginosa* showed mainly moderate to good activity against the microbial pathogens evaluated. Aqueous extract has showed low oral toxicity on rats since no animal death was detected at a dose of 2000 mg/kg. However, the extract promoted significant modifications of biochemical, hematological parameters and histopathological analysis showed alterations. These results indicate that caution is required regarding its consumption. *Acmella uliginosa* seems to be of particular interest for future investigations due to the toxicity of the aqueous extract but also because it is consumed by the population. Harvesting must be done in other geographical areas of Benin to reassess the toxicity.
